# Developing TeroENZ and TeroMAP modules for the terpenome research platform TeroKit

**DOI:** 10.1093/database/baad020

**Published:** 2023-05-18

**Authors:** Nianhang Chen, Rong Zhang, Tao Zeng, Xuting Zhang, Ruibo Wu

**Affiliations:** School of Pharmaceutical Sciences, Sun Yat-sen University, Guangzhou 510006, China; School of Pharmaceutical Sciences, Sun Yat-sen University, Guangzhou 510006, China; School of Pharmaceutical Sciences, Sun Yat-sen University, Guangzhou 510006, China; School of Pharmaceutical Sciences, Sun Yat-sen University, Guangzhou 510006, China; School of Pharmaceutical Sciences, Sun Yat-sen University, Guangzhou 510006, China

## Abstract

Terpenoids and their derivatives are collectively known as the terpenome and are the largest class of natural products, whose biosynthesis refers to various kinds of enzymes. To date, there is no terpenome-related enzyme database, which is a desire for enzyme mining, metabolic engineering and discovery of new natural products related to terpenoids. In this work, we have constructed a comprehensive database called TeroENZ (http://terokit.qmclab.com/browse_enz.html) containing 13 462 enzymes involved in the terpenoid biosynthetic pathway, covering 2541 species and 4293 reactions reported in the literature and public databases. At the same time, we classify enzymes according to their catalytic reactions into cyclase, oxidoreductase, transferase, and so on, and also make a classification according to species. This meticulous classification is beneficial for users as it can be retrieved and downloaded conveniently. We also provide a computational module for isozyme prediction. Moreover, a module named TeroMAP (http://terokit.qmclab.com/browse_rxn.html) is also constructed to organize all available terpenoid enzymatic reactions into an interactive network by interfacing with the previously established database of terpenoid compounds, TeroMOL. Finally, all these databases and modules are integrated into the web server TeroKit (http://terokit.qmclab.com/) to shed light on the field of terpenoid research.

**Database URL** http://terokit.qmclab.com/

**Key points**

- Terpenoids represent the largest family of natural products and exhibit diverse biological activities due to their complex structures, which are generated by terpenoid synthases, cytochrome P450 monooxygenases, UDP-glycosyltransferases, and other enzymes.
- A comprehensive terpenoid enzyme database called TeroENZ is collected, and a visualizable module called TeroMAP is developed to link terpenoid molecules and enzymes.
- TeroENZ and TeroMAP are deployed in the terpenome research platform TeroKit to facilitate gene clusters and enzyme mining, enzyme design, metabolic engineering and new natural product discovery for the community of terpenome research.

## Introduction

**Figure 1.**
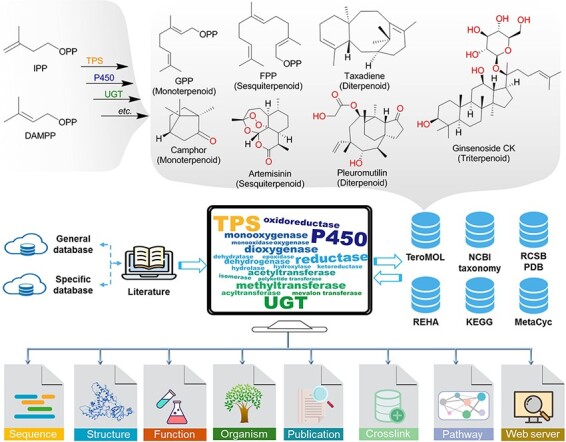
The brief biogenesis for terpenoids and the database resources for constructing the terpenome research platform TeroKit. The protocol of data collection is provided in Scheme S1.

Terpenoids, the structurally diverse and quantitatively largest class of natural products, are biosynthesized initially from two essential isoprene units, isopentenyl diphosphate (IPP) and dimethylallyl diphosphate (DMAPP) (Figure 1), and then followed by a series of chemical transformations conducted by various enzymes such as terpenoid synthases (TPSs—enzymes catalyzing isoprene coupling and cyclization reactions) to construct the essential carbon skeleton and cytochrome P450 monooxygenases (P450) and UDP-glycosyltransferases (UGTs) for its post-modification. Finally, the structural diversity of terpenoids is gained from those enzyme catalytic reactions including cyclization, oxidation, glycosylation, methylation, acylation and isomerization (1–3). As recently reported in the terpenoids database TeroMOL (4), there are a total of >150 000 terpenoids and their derivatives (together named terpenome) discovered in nature. According to the number of isoprene units, terpenoids are usually classified as hemiterpenoids, monoterpenoids, sesquiterpenoids, diterpenoids, sesterterpenoids, triterpenoids and so on. The so-called meroterpenoids, which are partly derived from terpene biosynthetic pathways, are further classified as polyketide-terpenoids and non-polyketide-terpenoids (5). The remarkable chemical diversity of terpenoids brings broad bioactivity to applications in disease therapy, fragrance, flavor, cosmetics and biofuel industries. For instance, camphor oil (6) and pleuromutilin (7) have been demonstrated to possess good anti-insect activity. Artemisinin is a well-known antimalarial drug (8). Taxadiene (9), celastrol (10) and glycoside ginsenoside compound K (11) are used as precursors for the chemical synthesis of notable drugs. Therefore, terpenoids have attracted extensive attention of scientists in pharmacy, chemistry, biology and other fields (12).

In this context, the development of terpenoid databases is an urgent demand, and thus, we constructed a data-driven webserver called TeroKit (13). To date, TeroKit has implemented a series of computational tools and a database of terpenoid compounds named TeroMOL (4), which contains around 180 000 terpenome molecules (updated December 2022) annotated with reported chemical structures, biological sources and bioactivity. However, data on the aforementioned essential enzymes (TPS, P450 and UGT) involved in terpenoid metabolism and their corresponding enzymatic reactions have not been gathered, which seriously hampered the practical use of TeroKit. As is well known, the metabolic pathways of terpene biosynthesis are efficiently pursued by searching for metabolic gene clusters in the genome of specific species using TPS and P450 (14). The mechanism decipherment of enzymatic reactions combined with the directed evolution promotes the biosynthesis of terpenoids (15). In addition, these studies could benefit from bioinformatics tools such as high-throughput sequencing, structure prediction and sequence alignment. Nevertheless, it is difficult to eliminate the large gap between limited annotation and massive sequences of terpenoid enzymes among those available databases. To date, there are some comprehensive databases with large collections of enzymes, but the accurate annotation and refined classification of the enzymes are lacking in most cases, and thus, these data are often unfriendly for specific studies of terpenoids. Recently, the plant-specialized terpenoids TPS enzymes database (Terzyme) (16), the plant-triterpene biosynthesis database (TriForC) (17) and the plant P450-dedicated databases (such as PCPD) (18, 19) have been developed to solve this dilemma. However, a comprehensive database of terpenome-related enzymes has not yet been constructed.

In this work, we developed a comprehensive database called TeroENZ and a visualizable module called TeroMAP to gather all experimentally available and computationally predictable information (such as enzyme identifiers, reactions, structures and organisms) for those enzymes involved in the terpene biosynthesis. Then, the two modules, TeroENZ and TeroMAP, are integrated into a common platform, TeroKit, with the previously established database and computational modules, to facilitate gene clusters and enzyme mining, enzyme design, metabolic engineering and new natural product discovery for the community of terpenome research.

## Materials and methods

As shown in Figure 1, terpenoid-related TPS, P450, UGT and other enzymes were retrieved from general enzyme databases, such as UniProt (20) and National Center for Biotechnology Information (NCBI) RefSeq (20), by fuzzy search. Then, the non-terpenoid–related items were manually removed according to the available annotations (such as reaction and function information). For items with ambiguous annotations, the original literature was retrieved and manually checked, which eventually led to the collection of 4894 entries. In addition, ∼1000 enzymes from the reported specific databases (Supplementary Table S1) were integrated into the collection.

Enzymatic reactions were also collected from MetaCyc (21), Kyoto Encyclopedia of Genes and Genomes (KEGG) (22) and Rhea (23) databases, and terpenoid-related reactions were extracted by the following process: for each product of the specific reaction, the most similar substrate evaluated by molecular fingerprint was kept to decompose the reaction into mono-substrate and mono-product pairs. Then, all the reaction pairs containing terpenoids from TeroMOL as either substrate or product were extracted, and all the structures were integrated into TeroMOL after they were manually checked. That process was repeated until no new reaction pair was extracted. The UniProt entries annotated for terpenoid reactions were collected and integrated into the above enzyme database, as shown in Figure 1.

Finally, all enzymes were merged and deduplicated according to the UniProt entries and sequence BLAST (for those without UniProt annotations), resulting in a total of 13 462 enzymes in the TeroENZ database. The functions of enzymes were annotated by the reaction pairs described earlier, which were classified into cyclization, oxidoreduction, transformation, and so on, based on the biosynthetic pathway of terpenoids and the enzyme commission (EC) classification. Since the reaction pairs link molecules and enzymes, they were organized as a network (named TeroMAP) that represents a ‘landscape’ of the metabolism of terpenoids. In addition, the organism information for all enzymes was collected from the original data sources and literature, followed by matching them to the NCBI taxonomy database (24) to get systematic annotations. Available crystal structures of 346 enzymes were collected from the RCSB Protein Data Bank  (25). The remaining 2931 enzymes were retrieved from the AlphaFold Protein Structure Database (26), while the remaining 185 enzyme structures were also predicted by the local AlphaFold2 program (27).

## Results

### Database content

**Figure 2.**
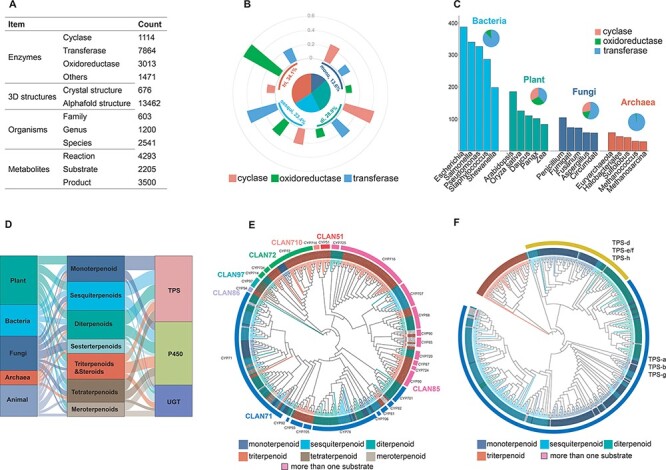
Database content. (A) The status of the recorded data for the construction of the TeroENZ database. (B) The pie chart shows the distribution of the enzymes that catalyze the four substrates and the histograms measured on the arcs represent the functional distribution of the enzymes (i.e. the values are normalized namely sum to 1 for each kind of enzyme). (C) Enzymes of different species sources were counted and the top five species were selected for display in bacteria, plants, fungi and archaea, respectively (histograms). All enzymes from these species were also classified according to function (pie charts). (D) The Sankey of species (left), substrate (middle) and enzyme (right). (E) Plant-derived P450 classified by family (evolutionary map). Multiple sequence alignment using muscle (34), evolutionary analysis with IQ-TREE (35), visualization with iTOL (36) and then a manual annotation. (F) Plant-derived TPS classified by function (evolutionary map), Outer ring: Class I, Class II. Inner ring: gray for angiosperms, black for gymnosperms and others.

To date, TeroENZ is the largest database of terpenoid enzymes (Figure 2A), comprising a total of 13 462 enzymes from 2541 species involving 4293 enzymatic reactions combined with 2205 substrates, 3500 products and the corresponding enzyme structures. All these enzymes are categorized by function as cyclases, oxidoreductases, transferases and others. The largest type of these transferases is the prenyltransferase, which is not discussed in the analysis due to the heterogeneity of its substrates. We also grouped all enzymes according to the catalytic substrate (Supplementary Table S2). Interestingly, a large proportion of the enzymes catalyze the reactions of hemiterpenes and the vast majority are transferases. As hemiterpenes are found in compounds such as flavonoids and phenylpropanoids, and they are also the essential units for catalyzing the synthesis of other terpenes, a similar phenomenon also occurs in heteroterpenes. To clarify the distribution of these three biggest classes of functional enzymes in the database, we selected only a total of 3075 enzymes from the widely studied monoterpenoids, sesquiterpenoids, diterpenoids and triterpenoids for analysis (Figure 2B). The results showed that cyclases were widely distributed in the biogenesis of monoterpenoids, sesquiterpenoids and diterpenoids, with the discovered sesquiterpenoid cyclases being particularly notable. Differently, oxidoreductases were also widely distributed, while they have been mainly reported for the modification of triterpene skeletons, possibly related to their larger number of structurally modifiable sites, as discussed in our previous study (4). The transferases are widely involved in the modification of diterpenes. In addition, we also selected a total of 11 085 enzymes and analysed their distribution among bacteria, plants, fungi and archaea (Figure 2C). The results showed that the largest number of enzymes were identified from the bacterial origin, followed by plants. Interestingly, transferases for terpenoid modifications are most prevalent in bacteria and archaea, while the three types of terpenoid enzymes identified in plants and fungi have a more balanced distribution.

It should be pointed out that the majority of transferases are found in bacteria, while for TPS, P450 and UGT, as summarized in Supplementary Table S3, only ∼31.42% of them are found in bacteria, most of them come from plants, fungi and archaea (86, 78 and 72%, respectively). Therefore, 1969 of the three famous terpenoid enzymes (in total, 6554 for TPS, P450 and UGT) are used for phylogenetic analysis, as summarized in Figure 2D; the substrates of TPS are mainly monoterpenoids, sesquiterpenoids and diterpenoids, and their main source is the plant. Differently, most of the substrates of P450 are triterpenoids and steroids , and they are mainly from plant and animal sources. Interestingly, UGT has the lowest amount and shows that the main substrates are meroterpenoids, triterpenoids and sesterterpenoids. It is noteworthy that these sesterterpenoids are mostly discovered in fungi and mostly have long-chain linear structures, whereas most of the UGT catalyzes the modification of the non-terpene structural motif of meroterpenoids.

Manually labeling these key enzymes (TPS, P450 and UGT) can help predict the catalytic function of others based on sequence similarity. Therefore, we constructed phylogenetic trees for each of them. Plant-derived P450 and TPS are shown as examples (see Figures 2E and F, respectively). The results showed that a total of 387 P450s were distributed among 7 clans with 26 families, where the clan71 and clan85 are the two largest clans. The clan71 mediated the biosynthesis of various terpenoids, particularly CYP71. CYP76, CYP88, CYP714, CYP720 and CYP725 were widely reported as they were involved in the biosynthesis of diterpenoids (28). For the clan85 (except for CYP725 and CYP720), CYP51, CYP72, CYP93, CYP94, CYP705, CYP710 and CYP734 are all involved in the biosynthesis of triterpenoids (29). However, the P450s are sporadically distributed in different families for monoterpenoids, sesquiterpenoids and others. Besides, it should be noted that CYP82, CYP85, CYP90 and CYP92 catalyze more than one type of substrate. Unlike P450, the 452 TPS enzymes could be clearly classified based on the substrate categories. The plant-derived TPS can be classified into Class I and Class II according to their conserved motifs. Substrates of Class I and Class II terpenoid cyclases are monoterpenoids/sesquiterpenoids and triterpenoids, respectively, while the terpenoid cyclases involved in diterpenoid biosynthesis are generally Class I or Class II (30). Interestingly, the vast majority of terpene cyclases are derived from angiosperms and a small number from gymnosperms, mosses, liverworts and ferns. In the case of diterpenes, for example, some of the diterpene cyclases derived from gymnosperms are bifunctional, as well as not containing the DxDD motif. In addition, the classification of UGT and other sources of P450 is shown in the supporting information (Supplementary Figures S1–S4).

### Web interface

**Figure 3.**
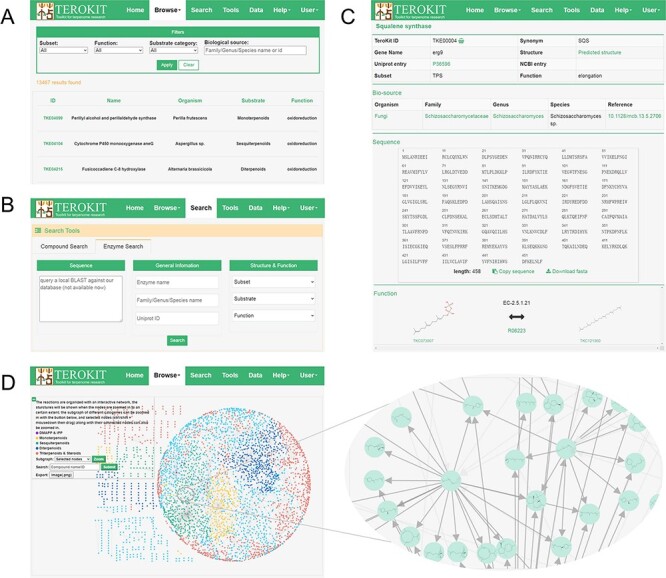
Demonstration of the web interface of TeroKit. (A) Browse, (B) search and (C) enzyme page for the use of TeroENZ, and (D) the browse page of TeroMAP.

TeroENZ is deployed on our previously developed web server TeroKit (http://terokit.qmclab.com). Users can browse all terpenoid enzymes with some custom-defined filter conditions (Figure 3A). As shown in Figure 3B, users can also search for enzymes based on more specific information, such as enzyme name and UniProt entry. Besides, a local BLAST (31) is also provided, by which similar enzymes can be retained based on the input sequence. The enzyme page (Figure 3C) shows basic information, including names, external links, structure (click to redirect to the structure sketcher) and functional classification. The organism with systematic annotations such as family, genus and species is also given, with each containing an external link. The sequence is also displayed and can be copied or downloaded directly, followed by the structure sketcher, in which the enzyme is rendered as a cartoon. The function of an enzyme is represented by the reaction pairs, and the molecules are linked with the built-in TeroMOL and the EC number, as well as the external links including MetaCyc, KEGG and Rhea. Moreover, TeroMAP is constructed as a special module to organize all available terpenoid enzymatic reactions in an interactive network (Figure 3D), where nodes represent the molecules and edges represent the reactions. Both node and edge are clickable to redirect to the corresponding molecule page or enzyme page. A sub-network can be zoomed according to the category of substrate or the selected nodes. A majority of molecules (3197 out of 3740) are connected to form the main network (see the circular layout in Figure 3D), with the rest being scattered around. In order to construct a complete terpenoid biosynthesis network, the previously deep learning-based retro-biosynthesis prediction tool, BioNavi-NP (32), is integrated as a module in the tool page and can be used to find the potential biosynthetic pathways for the scattered nodes or any molecules in TeroMOL as long as the users need. In addition, a biosynthetic mechanism prediction module for sesquiterpene is provided, based on our previously developed bio-inspired terpenoid generation strategy (33) with metadynamics and deep learning, by which users can explore a plausible terpenoid reaction network originated from any concerned carbocation (an example is presented in Supplementary Figure S5).

## Summary and perspectives

**Figure 4.**
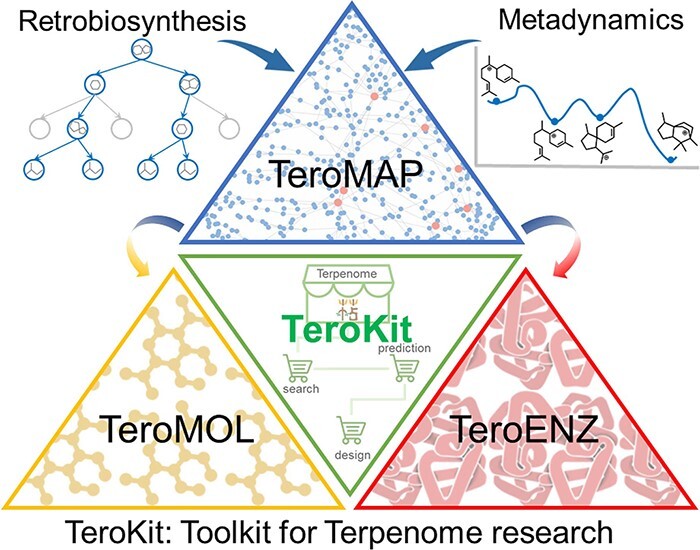
Schematic diagram of the terpenome research platform.

In this work, we have made a major update to a comprehensive database assembly TeroKit, which combined the previously developed TeroMOL with the current work on TeroENZ and TeroMAP. As shown in Figure 4, we believe that this user-friendly web service platform TeroKit will advance terpenome research by fully collecting, integrating, meticulously classifying and annotating data gathered from the literature and public databases.TeroKit is also available to share all information about terpenoid-related enzymes including data browsing and data retrieval. The development of TeroENZ enables interoperability between the terpenome and enzymes through reaction information in combination with TeroMOL, and in this way, the terpene metabolic network TeroMAP is subsequently constructed to conveniently visualize the biosynthetic networks. Moreover, all these databases and modules are integrated into a unified platform TeroKit (http://terokit.qmclab.com/) to accelerate terpenome research, including enzyme mining, metabolic engineering and new natural product discovery.

The maintenance and sustainability of these databases is a key concern, with a mature website framework in place to facilitate the regular uploading of new data. We are also keen for terpenome-related enzyme data to be supplemented by emails from researchers, and we have dedicated staff to review and archive these data annually. In the future, more computational modules will be integrated into this highly assembled terpenome research platform TeroKit.

## Supplementary Material

baad020_Supp

## Data Availability

All the data used in this manuscript are collected from the public database and can be free accessed or downloaded by visiting our website (http://terokit.qmclab.com/data.html).
