# Supplementary material for: Developing TeroENZ and TeroMAP modules for the terpenome research platform TeroKit
Source: Database (Oxford). 2023 May 18;2023:baad020. doi: 10.1093/database/baad020 (PMC10380177; doi:10.1093/database/baad020)
Supplement: baad020_Supp [file baad020_supp.zip › suppl_data/Supplementary material..docx]

**Developing TeroENZ and TeroMAP modules for the terpenome research platform TeroKit**

Nianhang Chen^#^, Rong Zhang^#^, Tao Zeng^#^, Xuting Zhang, Ruibo Wu*

School of Pharmaceutical Sciences, Sun Yat-sen University, Guangzhou, 510006, China

^#^ The authors wish it to be known that the first three authors should be regarded as joint First Authors

* To whom correspondence should be addressed. Email: [wurb3@mail.sysu.edu.cn](mailto:wurb3@mail.sysu.edu.cn)

Table S1-S3

Table S1. Database resources used for TeroENZ construction

Table S2. Information on terpene enzymes with different substrates in the TeroEnz database

Table S3. Distribution of TPS/P450/GTs in different species

Scheme S1

The protocol of data collection

Figure S1-S4

Figure S1. UGT phylogenetic tree

Figure S2. P450 phylogenetic tree in fungi

Figure S3. P450 phylogenetic tree in animal

Figure S4. P450 phylogenetic tree in bacteria

Table S1. Database resources used for TeroENZ construction

| **Database** | **Descriptions** | **Webpage** |
| --- | --- | --- |
| Uniprot | General enzyme database | https://www.uniprot.org/ |
| NCBI RefSeq | General enzyme database | https://www.ncbi.nlm.nih.gov/ |
| CAZY | Glycosyltransferase-specific database | http://www.cazy.org/ |
| GTDB | Glycosyltransferase-specific database | https://www.biosino.org/gtdb/ |
| The Plant Cytochrome P450 Database | P450-specific database | https://erda.dk/public/vgrid/PlantP450/ |
| Plant Cytochrome P450 Database (PCPD) | P450-specific database | http://p450.biodesign.ac.cn/ |
| TriForC Database | Triterpenoid biosynthesis-specific enzyme database | http://bioinformatics.psb.ugent.be/triforc/ |
| MetaCyc | Metabolic reaction database | https://metacyc.org/ |
| RHEA | Metabolic reaction database | https://www.rhea-db.org/ |
| KEGG | Metabolic reaction database | https://www.genome.jp/kegg/ |
| TeroMOL | Terpenoid molecule database | http://terokit.qmclab.com/ |
| NCBI taxonomy | Database of organism classification and nomenclature | https://www.ncbi.nlm.nih.gov/taxonomy |
| RCSB Protein Data Bank | Experimental 3D structures database for biological molecules | https://www.pdbus.org/ |
| AlphaFold Protein Structure Database | Predicted 3D structures database for biological molecules with AlphaFold2 | https://www.alphafold.ebi.ac.uk/ |

Table S2. Information on terpene enzymes with different substrates in the TeroEnz database

| Type of substrates | Type of information | | | | | | |
| --- | --- | --- | --- | --- | --- | --- | --- |
|  | No. of enzymes | No. of reactions | No.of  substrates | No. of  crystal structures | No. of cyclases | No. of transferases | No. of oxidoreductases |
| monoterpenoid | 424 | 331 | 160 | 98 | 173 | 138 | 108 |
| sesquiterpenoid | 727 | 558 | 181 | 104 | 297 | 261 | 163 |
| diterpenoid | 899 | 608 | 301 | 145 | 153 | 304 | 432 |
| sesterterpenoid | 174 | 52 | 31 | 6 | 9 | 157 | 8 |
| triterpenoid&steroid | 1048 | 1295 | 635 | 283 | 77 | 199 | 761 |
| tetraterpenoids | 372 | 287 | 141 | 15 | 7 | 253 | 93 |
| meroterpenoid | 1018 | 538 | 396 | 42 | 4 | 702 | 312 |
| Others(hemiterpenoid) | 5627 | 624 | 376 | 140 | 6 | 4009 | 588 |

Table S3. Distribution of TPS/P450/GTs in different species

| Species | Sum of all enzyme | Sum of (TPS/P450/GTs) | Proportion (%) |
| --- | --- | --- | --- |
| Plant | 1853 | 1594 | 86.02 |
| Bacteria | 7940 | 2495 | 31.42 |
| Fungi | 1477 | 1160 | 78.54 |
| Archase | 887 | 638 | 71.93 |
| Animal | 1083 | 454 | 41.92 |

Scheme S1


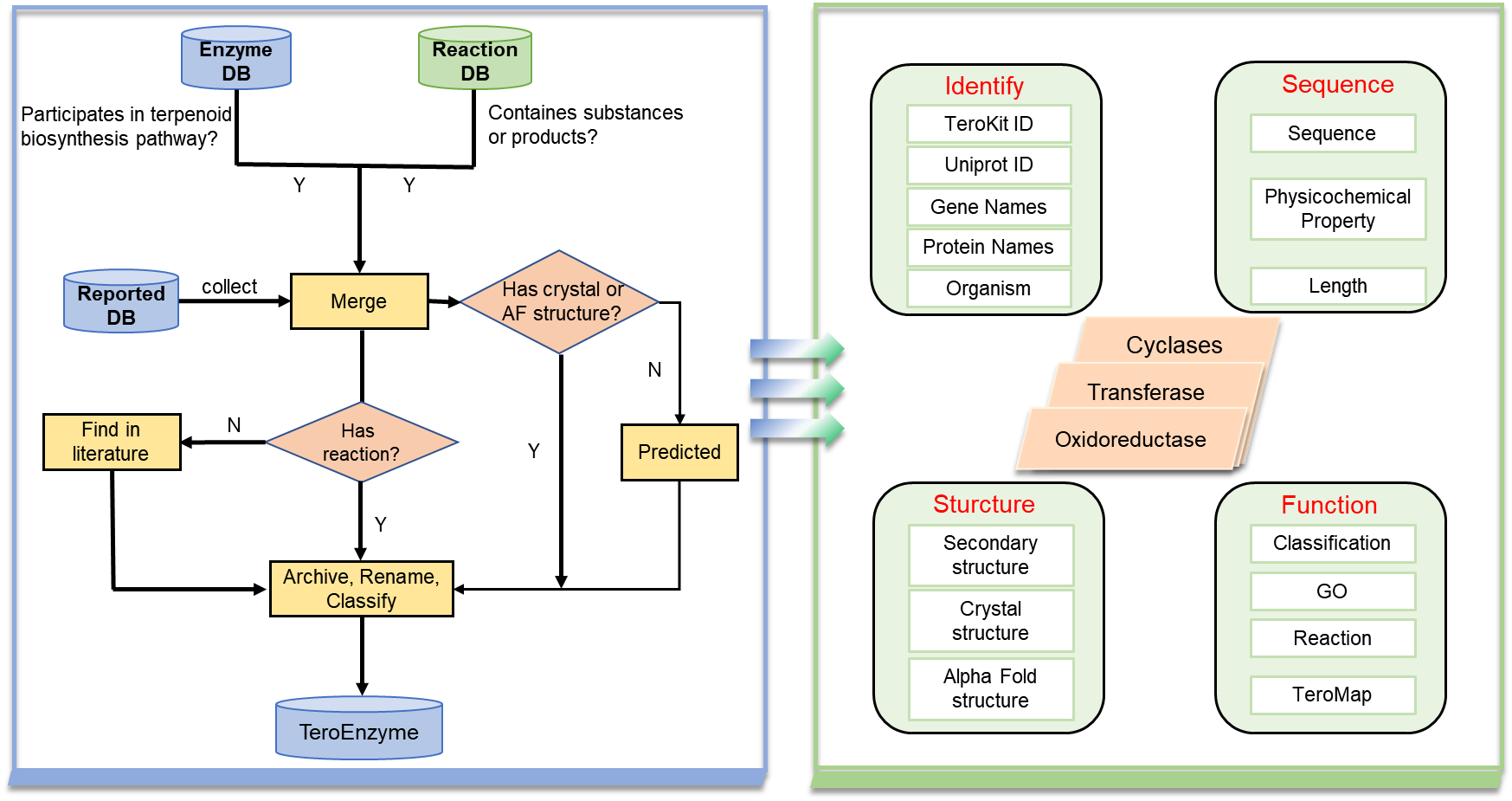


The protocol of data merging and archiving, the left one shows the data processing flow, the right one shows the basic information about TeroENZ.

Figure S1


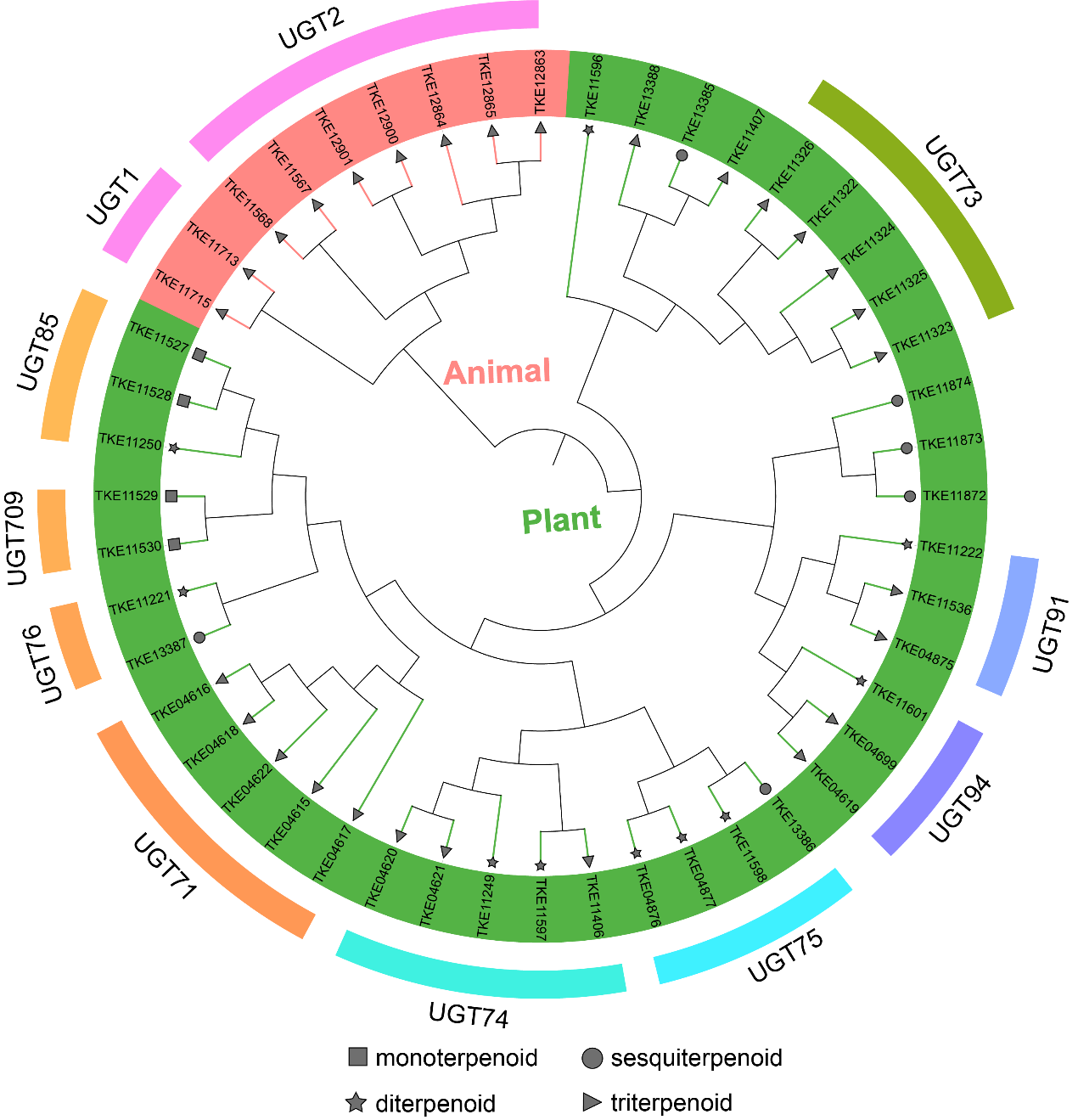


UGT phylogenetic tree. Green for plants, red for animals, squares, circular pentagrams, triangles for catalytic substrates and outer circles for families to which UGT belongs.

Figure S2


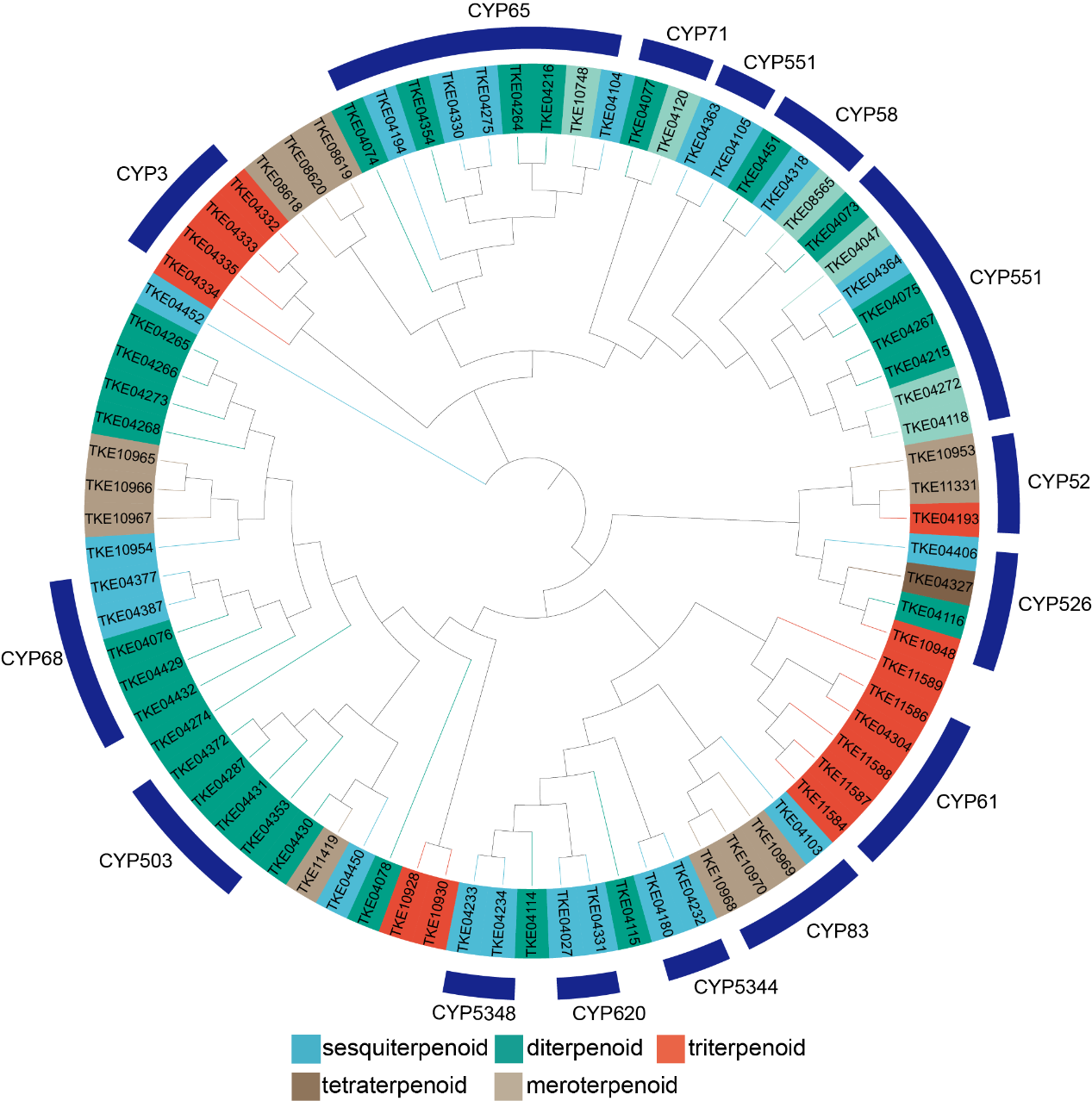


P450 phylogenetic tree in fungi.

Figure S3


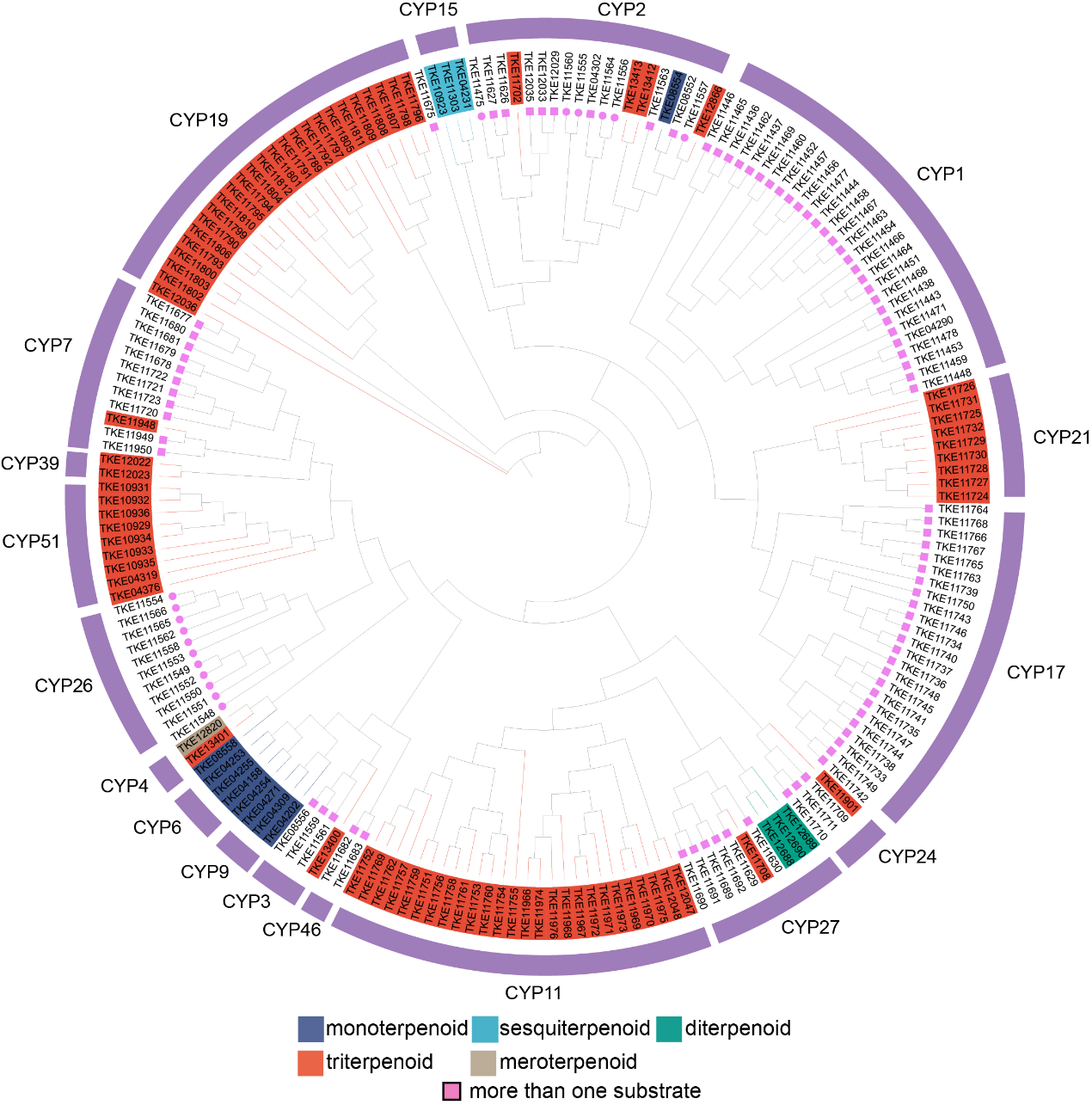


P450 phylogenetic tree in animal.

Figure S4


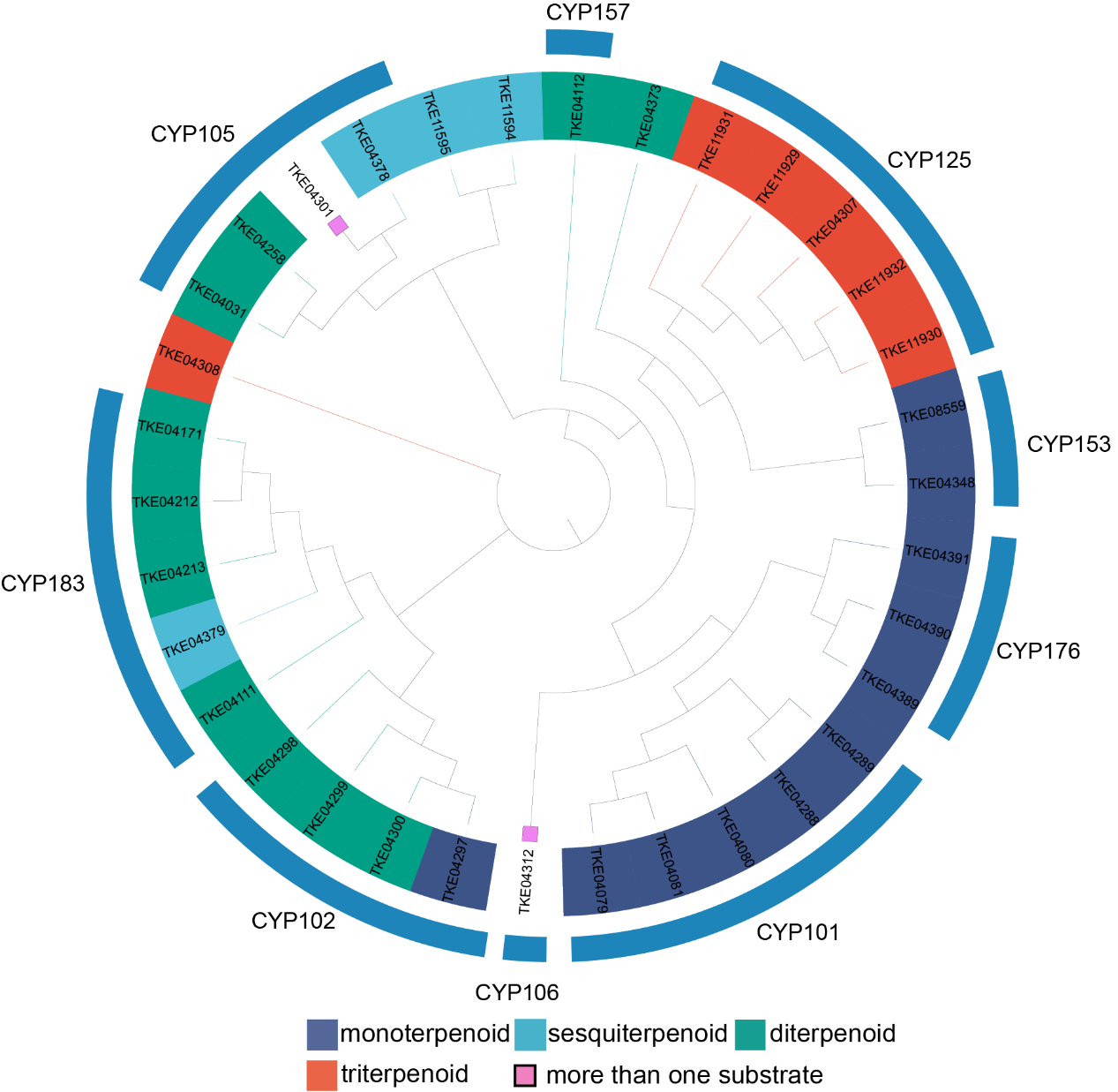


P450 phylogenetic tree in bacteria.
